# The effect of prolonged holding time on the mechanical property and microstructural property of lithium disilicate glass-ceramic

**DOI:** 10.1007/s10856-022-06693-0

**Published:** 2022-10-03

**Authors:** Feng Lin, Bin Wang, Yanmei Zhang, Shuigen Li, Qiufang Zhang, Yin Xiao, Qiliang Zuo

**Affiliations:** 1 Stomatological Hospital of Xiamen Medical College, Xiamen Medical College, Xiamen, PR China; 2 Xiamen Key Laboratory of Stomatological Disease Diagnosis and Treatment, Xiamen, PR China; 3 Engineering Research Center of Fujian University for Stomatological Biomaterials, Xiamen, PR China; 4 State Key Laboratory of Physical Chemistry of Solid Surfaces, College of Chemistry and Chemical Engineering, Xiamen University, Xiamen, China; 5 Institute of Health and Biomedical Innovation, School of Chemistry, Physics, Mechanical Engineering, Queensland University of Technology, Brisbane, QLD Australia; 6 Australia-China Centre for Tissue Engineering and Regenerative Medicine, Brisbane, QLD Australia

## Abstract

Repeat firing produces uncertainty about stabilizing lithium disilicate glass-ceramic (LDGC) material properties, even though prolonged holding time can enhance the mechanical property of LDGC during a single firing cycle. However, the effect of prolonged holding time and repeat firing on the mechanical property and microstructure of LDGC is not fully understood. In the present study, three groups of LDGC material were created: (i) extension of holding time (7 vs. 14 vs. 28 min) at 780–800 °C; (ii) holding time extension (7 vs. 14 min) and dual sintering at 800–820 °C, respectively; (iii) dual sintering with prolonged holding time (7 vs. 14 min) at 820–840 °C. The nano-indenter test revealed that prolonged holding time (14 and 28 min) promoted the enhancement of LDGC hardness and Young’s modulus. X-ray photoelectron spectroscopy, X-ray diffraction and Fourier transform infrared spectroscopy confirmed that prolonged holding time increased and stabilized LD phase in LDGC, as well as induced residual compressive stress. Scanning electron microscopy showed that prolonged holding time increased LD crystal grains homogeneously and facilitated LDGC to form dense interlocking structure without enlarging crystal size grains significantly. In contrast, LDGC that dual sintered alone at 820–840 °C possessed inferior mechanical properties, coupled with heterogeneous crystal phases, residual tensile stress, and melted crystals grains in the porous microstructure. Interestingly, these deteriorated properties of LDGC caused by dual sintering alone could be counteracted by prolonging the holding time. Nevertheless, the LDGC materials displayed an excellent biocompatibility throughout the study. This study identified that prolonged holding time during repeated firing cycles stabilized LD phase and crystal grain size of LDGC, thus enhanced the mechanical properties, which provided a new insight to extend the repeat fired restoration longevity of LDGC.

**Figure:**
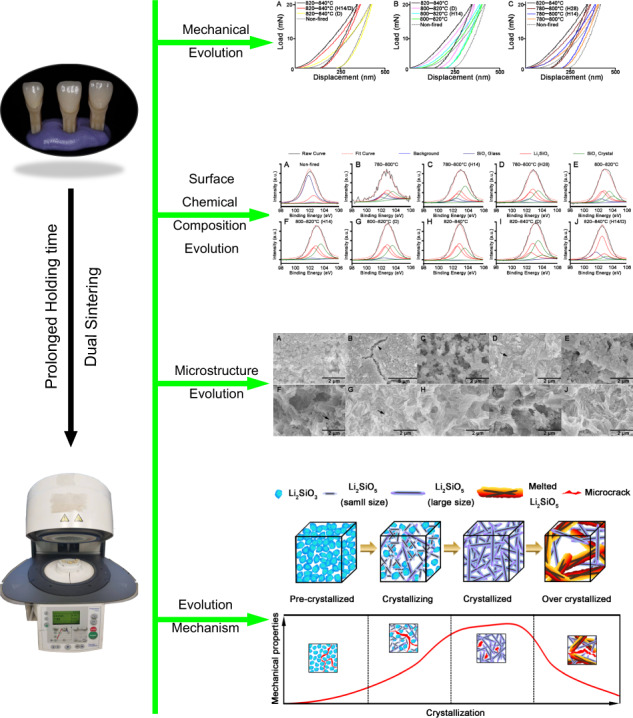
Graphical abstract

Graphical abstract

## Introduction

The inception of “digital dentistry” and advances in computer-aided design (CAD) and computer-aided manufacturing technology has revolutionized dentistry. Lithium disilicate glass ceramic (LDGC) material was introduced to maximize the quality and efficiency of prostheses manufacture, and showed exceptional mechanical and esthetic properties [1, 2]. However, the LDGC prostheses sometimes suffer from additional adjustment and dual sintering to satisfy the clinical reasons such as the limit of the esthetic outcome or a deficient proximal contact, which in turn produce uncertainty to the stabilization of material properties. For instance, repeat firing was demonstrated to have no effect on the “nano-hardness” of LDGC glass [3], but Meng et al. reported ceramic plates that received one firing cycle displayed a higher fracture toughness than those that received multiple firing cycles [4]. Obviously, the mechanical properties of LDGC materials were apt to be diminished during multiple firing cycles, by which the restoration longevity of the LDGC could be reduced. In fact, there is a distinct lack of clinical literature discussing the solution to alleviate the deterioration of LDGC materials properties when the repeated firing cycles is unavoidable.

For LDGC materials, the conventional wisdom is that excellent mechanical properties match “ideal” microstructures which have been formed by “interlocked” lithium disilicate (LD) grains, and show the best balance of the conflicting effects of crystallinity degree, grain size, and residual stress [5]. To achieve this target, better understanding of the impact of crystallization on the mechanical properties and corresponding phase transformation of LDGC at various thermal treatment conditions is paramount. Lien et al. reported that extension of the holding time contributed to reinforcement of the mechanical properties of a LDGC material, but the mechanism was not elucidated [6]. Evidence also showed that the extension of holding time facilitated the crystal phase transformation at initial heating stage and had less influence on the crystal size of LDGC at final stage [7]. Efforts that were made to study the details of the sintering conditions confirmed that temperature was critical to the material properties of LDGC as well [8]. Hence, whether a prolonged holding time could improve the mechanical properties of re-sintered LDGC materials and corresponding mechanism are worth more exploring.

According to literatures, X-ray diffraction (XRD) shows good sensitivity for identifying phase transformations [6, 9]. However, XRD cannot be used to analyze the small discrepancies among mature LDGC materials heated at high temperature because the LD phase becomes the predominant phase in these materials [10, 11]. Therefore, the mechanical and microstructural evolution of LDGC under altered thermal conditions is still not fully known. Recently, X-ray photoelectron spectroscopy (XPS) was used to survey four highly sintered LDGC materials, and revealed significant chemical alterations on the sample surface [12]. Besides, Ghayebloo et al. investigated ZrO_2_-bearing LDGC materials fabricated by pressure-less sintering and spark plasma sintering, and minor alterations of chemical bonds were identified by Fourier transform infrared (FTIR) spectroscopy [13]. These studies provided us potential tools to reveal the subtle alteration of the LDGC microstructure.

With these premises in mind, we investigated the effect of the holding time, and dual sintering on the mechanical properties and corresponding phase transformation of an LDGC block. Nanoindentation, XRD, XPS, FTIR spectroscopy, and field emission scanning electron microscopy (FESEM) were employed concomitantly to investigate the mechanical properties and microstructure in detail, the biocompatibility of LDGC materials with different treatments were evaluated as well. In this way, information on the heating schedule could be used to optimize the mechanical properties of LDGC prostheses for dental application.

## Methodology

### Sample preparation

Commercially available dental-grade LDGC blocks (IPS emax CAD LT, C2, shade A2; Ivoclar Vivadent, Schaan, Liechtenstein) were machined into slides (approximately 12 × 10 × 1 mm^3^) by a liner precision saw (Isomet 4000; Buehler, Uzwil, Switzerland). Then, they were polished gradually by silicon-carbide paper of 600-, 800-, 1000-, 1200-, 1500- and 2000 girt (Golden Sun Abrasive, Dongguan, China) under running water at 500 rpm on a polishing machine (YMP-1A; Metallurgical Equipment, Shanghai, China). Then, the specimens were rinsed with deionized water (A2S-05-BE; AquaPure, Fresno, CA, USA) and stored dry before processing and tests.

### Heating procedure

According to manufacturer recommendations, a two-stage sintering schedule was used as a standard protocol to ensure transformation from a partially crystallized microstructure to fully crystallized microstructure in LDGC blocks.

Eight unique two-stage sintering schedules (Table 1) were set up to evaluate the effect of the heating parameter on evolution of the properties of the material. A non-fired specimen and 820–840 °C group (recommended schedule) were the control groups. Groups with an altered final target temperature could reveal the influence of the sintering temperature on the microstructure and mechanical evolution of the material. Groups labeled “H14” (i.e., holding time was 14 min) and “H28” permitted investigation of the impact of a prolonged holding time. Dual sintering (D) groups were sintered twice, which imitated the process of ceramic re-sintering due to reworking of prostheses. Among these groups, the door-closing time, furnace standby temperature, heating rate, and the first holding time were kept constant.

**Table 1:** Two-stage heating schedules

Group	Non-fired	780–800 °C		780–800 °C (H14)		780–800 °C (H28)		800–820 °C		800–820 °C (H14)		800–820 °C (D)		820–840 °C		820–840 °C (D)		820–840 °C (H14/D)	
Stage	_	1	2	1	2	1	2	1	2	1	2	1	2	1	2	1	2	1	2
B (°C)	–	403		403		403		403		403		403		403		403		403	
S (min)		6		6		6		6		6		6		6		6		6	
t (°C/min)		90	30	90	30	90	30	90	30	90	30	90	30	90	30	90	30	90	30
T (°C)		780	800	780	800	780	800	800	820	800	820	800	820	820	820	820	840	820	840
H (min)		0.10	7	0.10	14	0.10	28	0.10	7	0.10	14	0.10	7	0.10	7	0.10	7	0.10	14
V_1_ (°C)		550	780	550	780	550	780	550	800	550	800	550	800	550	820	550	820	550	820
V_2_ (°C)		780	800	780	800	780	800	800	820	800	820	800	820	820	840	820	840	820	840

*B (°C)* Furnace standby temperature, *S (min)* furnace door closing time, *t (°C /min)* heating or ramp rate, *T (°C)* holding temperature, *V*_*1*_ *(°C)* vacuum-on temperature, *V*_*2*_ *(°C)* vacuum-off temperature, *H (min)* holding time, *H14* hold for 14 min, *H28* hold for 28 min, *D* dual sintering

### Nanoindentation

Nanoindentation testing was used to assess the Young’s modulus and hardness of the material. Load-controlled nanoindentation measurements were undertaken by a Nano Hardness Tester (NHT) nanoindenter (CSM Instruments, Peseux, Switzerland). A diamond Berkovich pyramidal tip was employed for all measurements under a trapezoidal loading function, and was calibrated using a fused silica standard. The constant loading rate and constant unloading rate were 40 mN/min, and the corresponding time was 30 s, respectively. The material was indented with a maximum load (*P*_max_) of 20 mN, which was followed by a pause time of 2 s. Hardness and Young’s modulus were calculated from the unloading segment of the load–displacement curve using the Oliver–Pharr method [14]. A Poisson ratio of 0.3 was assumed for computation of Young’s modulus. Ten sets of all groups were measured. Mean values for hardness and Young’s modulus were calculated for each specimen.

### Characterization of phase transformation

#### XPS

A scanning ESCA microprobe (Quantum 2000; Physical Electronics, Eden Prairie, MN, USA) with monochromatic Al Kα (1486.6 eV) irradiation was employed for XPS. Spectra were calibrated using the C 1 s (284.6 eV) peak of the hydrocarbon remaining in the XPS analysis chamber as a contaminant. All 10 slices of each group were analyzed randomly in five regions. Removal of the Shirley background was followed by element identification and processing of peak deconvolutions using Multipak V6.1 A (Physical Electronics) and XPSPEAK 41 (Casa Software, Teignmouth, UK). The full width at half-maximum for peak deconvolution was kept constant in 1.6 eV for Si2p. The Lorentzian–Gaussian ratio was fixed at 80%.

#### XRD

Slices were placed in the holder of an XRD system (Ultima IV; Rigaku, Tokyo, Japan) and scanned using Cu Kα X-rays with a step size of 0.02° in a 2θ range of 10–90°. Phase development analyses were undertaken by Jade 6 (MDI; https://www.icdd.com/mdi-jade/) as well for the reference data to enable interpretation of the XRD patterns obtained from Joint Committee on Powder Diffraction Standards (JCPDS) patterns. Residual stress measurements were conducted by PANalytical X-Pert Pro materials research diffraction (MRD) system (Philips X’pert PRO MRD, Almelo, Netherlands) using Cu Kα X-rays with a wavelength of 1.56 Å in a 2θ range of 68–72°. The lattice spacing was measured in 6Ψ tilts (0, 5, 15, 25, 35, 45°). The calculations were performed using “X’Pert Stress 1.1a” XRD software module.

#### FTIR spectroscopy

FTIR spectra were recorded with a Nicolet is50 system (Thermo Fisher, Waltham, MA, USA) with an attenuated total reflectance accessory in the range 400–4000 cm^−1^. The measurement error was ±0.1%. The number of scans was 32 at a resolution of 4 cm^−1^ in absorbance mode.

#### SEM

LDGC samples were etched with hydrofluoric acid (5 vol%) for 3 min and then rinsed with deionized water. Slices were coated with platinum and examined by FESEM using a S4800 system (Hitachi, Tokyo, Japan) operating at 10 kV for microstructural observation.

### Estimation of LDGC biocompatibility

#### Cell proliferation

A preosteoblast MC3T3-E1 cell line was obtained from the Cell Resource Center, Peking Union Medical College (the headquarters of the National Infrastructure of Cell Line Resource, NSTI). MC3T3-E1 cells were seeded on the sterile LDGC discs in a 12-well cell culture plate at a density of 5 × 10^4^ cells per well and cultured for 2 and 5 days. The α-MEM (Thermo Fisher Scientific, USA) supplemented with 10% FBS (PAN-Biotech GmbH, Germany) was refreshed every 2 days. A cell proliferation colorimetric assay was conducted with the Cell Counting Kit-8 (CCK-8, Enzo Life Sciences, USA) for 2 and 5 days, according to the manufacturer’s instructions. After 2 h reaction, 100 µl of the above medium containing CCK-8 solution was transferred into a 96 well cell culturing plate. Optical absorbance of reaction solution was recorded using a plate reader (Infinite F50, Tecan) equipped with a 450 nm filter.

#### Cell morphology

LDGC samples cultured with MC3T3-E1 cells for 5 days were fixed in 2.5% glutaraldehyde and 1.5% paraformaldehyde at 4 °C for 1 h. The specimens were then dehydrated in ascending grades of ethanol and tertiary butanol. After drying with 100% tertiary butanol, specimens were coated with sputtered gold for further SEM (FlexSEM 1000, Hitachi High Technologies corp., Japan) analysis.

### Statistical analyses

Data were analyzed with SPSS 22 (IBM, Armonk, NY, USA). One-way analysis of variance followed by Student–Newman–Keuls-*q* (SNK-*q*) tests were done for multiple comparisons at a significance level of *p* = 0.05.

## Results

### Evolution of mechanical properties

**Fig. 1:**
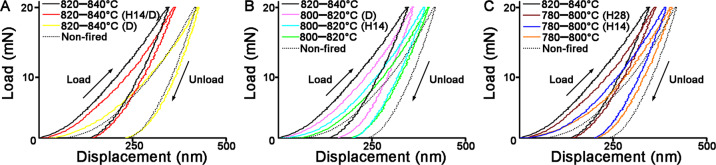
Load–displacement curves of LDGC materials heated under various thermal conditions. Non-fired and 820–840 °C groups were set as reference in each group to estimate the mechanical properties of LDGC materials that were treated at various temperature levels. **A** 820–840 °C; **B** 800–820 °C; **C** 780–800 °C. The loading curve was related to elastic deformation of the contact, while the unloading curve was response to the elastic recovery of the contact. Group with longer displacffement indicated a weaker mechanical properties

Figure 1 shows load–displacement curves. Upon indention, the non-fired group deformed much easier than the fired group, showing longer displacement at the same peak load (20 mN), while the 820–840 °C group displayed an optimum mechanical performance. Both a prolonged holding time and dual sintering had a significant impact on the mechanical properties of the LDGC block. As it is shown, the 820–840 °C (D) group suffered from a similar displacement which was close to the non-fired group, whereas 820–840 °C (H14/D) group merely displayed a slightly longer displacement than the 820–840 °C group (Fig. 1A). The groups for dual sintering and prolonged holding time at 800–820 °C presented a higher resistance to deformation than the group that did not undergo an additional process (Fig. 1B). In 780–800 °C groups, the displacement decreased significantly with an increasing holding time at the same peak load (Fig. 1C). Table 2 summarizes all the values for mean ± standard deviation for hardness and Young’s modulus. The result illustrated that, at 820–840 °C, dual sintering decreased the mechanical properties of LDGC, but a prolonged holding time stabilized the mechanical properties of LDGC. Both dual sintering and prolonged holding time promoted the mechanical properties of LDGC when the sintering temperature was below 820 °C.

**Table 2:** Average values for hardness and Young’s modulus of LDGC materials under different heating conditions

Group	Hardness (GPa)	Young’s modulus (GPa)
Non-fired	5.63 ± 0.14^a^	74.05 ± 1.06^α^
780–800 °C	5.58 ± 0.12^a^	83.50 ± 2.15^β^
780–800 °C (H14)	7.76 ± 0.10^b^	92.09 ± 0.80^γ^
780–800 °C (H28)	12.18 ± 0.52^c^	108.39 ± 1.15^δ^
800–820 °C	7.67 ± 0.18^b^	87.20 ± 1.46^ε^
800–820 °C (H14)	6.64 ± 0.26^d^	93.92 ± 2.21^γ^
800–820 °C (D)	15.64 ± 1.04^e^	104.30 ± 2.18^ζ^
820–840 °C	18.01 ± 1.20^f^	117.44 ± 1.85^η^
820–840 °C (D)	4.37 ± 0.66^g^	72.95 ± 1.86^α^
820–840 °C (H14/D)	11.99 ± 1.00^c^	113.95 ± 0.87^θ^

Values (mean ± SD) with superscripts a–g and α–θ at the same line had a significant difference (one-way ANOVA and SNK-*q* test, *P* < 0.05)

### Phase transformation under various thermal parameters

**Fig. 2:**
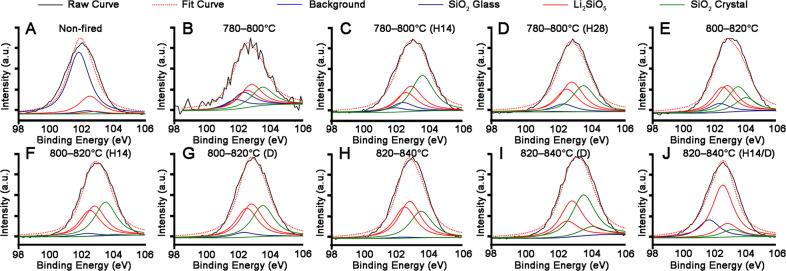
XPS showing high-resolution peaks for Si2p in LDGC materials heated under various thermal conditions. The details of deconvolution are listed in Table 3

The narrow-scan XPS spectrum of the Si2p region in each group was decomposed into three contributions (Fig. 2). The peaks located at 101.8–102.3 eV could be assigned to an amorphous SiO_2_ and pre-crystallized LM, in which silicon was bound to two oxygen atoms [12, 15]. The binding energies around 102.5–102.8 eV were due to the chemical environment related to the Q3 species of Si2p, which is a characteristic of LD [15, 16]. Broad peaks at 103.5–104 eV attributed to SiO_2_ crystals were signatures of Si^4+^, in which silicon can connect to four oxygen atoms [12, 15]. It was note that the position of the assigned peaks shifted from low binding energy to high level as the prolonged holding time and dual sintering were applied. Dual sintering promoted the generation of the peaks at 103.5–104 eV (Fig. 2I); however, prolonged holding time stabilized the peaks at 102.5–102.8 eV (Fig. 2J). The quantitative phase results of the corresponding peak deconvolutions (which were identified by peak-fitting procedures) are displayed in Table 3. The amount of Si2p B increased during an increase in temperature and the holding time, whereas the amount of Si2p A decreased, as shown in 780–800 °C, 780–800 °C (H14), 780–800 °C (H28), 800–820 °C, 800–820 °C (H14) and 820–840 °C groups. Dual sintering promoted the formation of Si2p B at 800–820 °C, but reduced the amount of Si2p B at 820–840 °C. In the 820–840 °C (H14/D) group, increases in the amount of Si2p B and Si2p A were identified, but the amount of Si2p C declined significantly.

**Table 3:** Peak positions for silicone of a LDGC material upon different treatments

Peak deconvolution	Peak position	Attribution	LDGC group (%)									
			Non-fired	780–800 °C	780–800 °C (H14)	780–800 °C (H28)	800–820 °C	800–820 °C (H14)	800–820 °C (D)	820–840 °C	820–840 °C (D)	820–840 °C (H14/D)
Si2p A	101.8–102.3	SiO_2_ glass	76.32	30.28	14.56	9.72	9.06	3.19	4.93	1.12%	0.01	19.49
Si2p B	102.5–102.8	Li_2_SiO_5_	23.68	47.74	47.47	60.35	52.28	59.96	63.33	71.04	51.21	72.41
Si2p C	103.5–104.0	SiO_2_ crystal	0	21.98	37.97	29.93	38.66	36.85	31.74	27.84	48.78	8.09

**Fig. 3:**
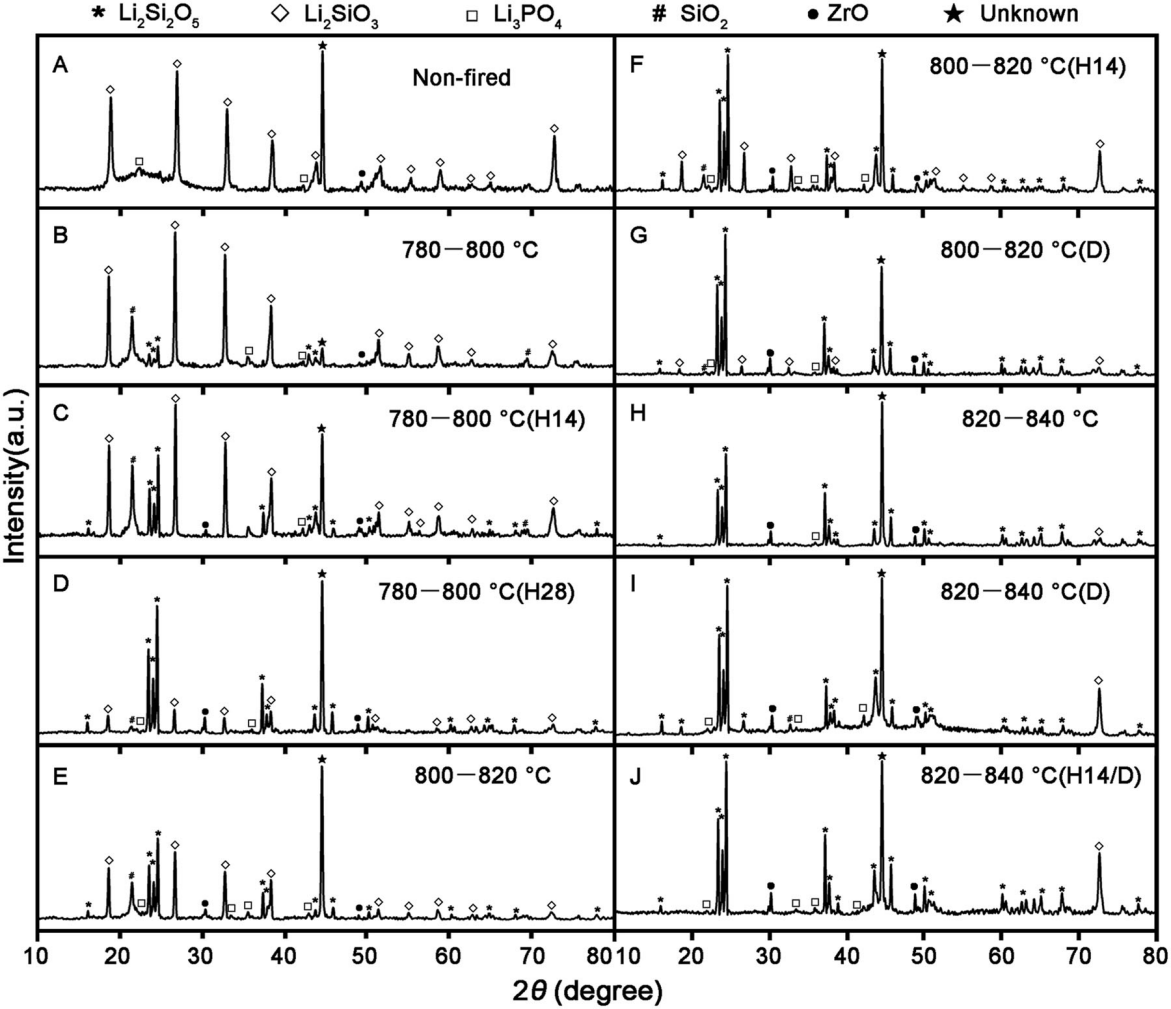
XRD patterns of LDGC materials heated under various thermal conditions

Phases of LDGC samples were qualitatively determined by XRD (Fig. 3). The major crystalline phase detected in the non-fired sample was LM (Fig. 3A). Some of the LD phase was found in the 780–800 °C group (Fig. 3B). Stronger peaks of LD could be identified if the holding time was doubled (Fig. 3C). Despite a sharp rise in LD-phase intensity (23.4°, 24°, and 24.4°) being detected when the holding time was 28 min, the LM phase remained (Fig. 3D). Multiple phases seemed to be distributed equally in the 800–820 °C group (Fig. 3E). The intensity of the LD phase was increased by extension of the holding time and dual sintering at 800–820 °C, whereas the intensity of the LM phase faded, especially in the 800–820 °C (D) group (Fig. 3F, G). A discernable peak, settled at 2θ values of 21.4° in 780–800 °C, 780–800 °C (H14), 800–820 °C, and 800–820 °C (H14) groups, could denote the presence of cristobalite. LD was the primary crystal phase in well-sintered samples fired by a standard sintering protocol (Fig. 3H). Irrespective of whether the holding time was prolonged, dual sintering led to the reappearance of LM-phase peaks, settled at 2θ value of 72.9°, in the LDGC material (Fig. 3I, J). However, the discrepancy between the 820–840 °C (H14/D) group and 820–840 °C (D) group was difficult to distinguish. We also discovered from the peaks that lithium orthophosphate and zirconia were indispensable components in the sintered groups. Furthermore, there was a permanent peak in all groups which we labeled “unknown”. Jade (MDI) revealed that the diffraction pattern of the unknown component (44.6°) could probably be attributed to a mixture of aluminum, cobalt and nickel, and was named Al_4_Co_3_Ni_3_ (JCPDS 46-1062). The average residual stresses were displayed in Table 4. The residual stresses calculated for non-fired, 780–800 °C (H28), 800–820 °C (D), 820–840 °C, and 820–840 °C (H14/D) groups were compressive, in which they were largest for 820–840° group and in the range −3 to −34.8 MPa for the other groups. However, the residual stresses calculated for 780–800 °C, 780–800 °C (H14), 800–820 °C, 800–820 °C (H14), and 820–840 °C (D) groups were tensile and in the range 2.7 to 27.5 MPa. The LDGC that was undergoing the phase transformation from LM to LD displayed residual tensile stress, as shown in 780–800 °C, 780–800 °C (H14), 800–820 °C and 800–820 °C (H14). The 780–800 °C (H28), 800–820 °C (D), and 820–840 °C, with well-crystallized LD presented residual compressive stress. Interestingly, dual sintering alone caused a residual tensile stress in LDGC material above 820 °C, while 820–840 °C (H14/D) groups possessed a residual compressive stress.

**Table 4:** Average values for residual stress of LDGC materials under various heating conditions

Group	Residual stress (MPa)
Non-fired	−3.3 ± 0.3
780–800 °C	2.7 ± 2.2
780–800 °C (H14)	8.1 ± 0.8
780–800 °C (H28)	−20.3 ± 2.0
800–820 °C	27.5 ± 2.3
800–820 °C (H14)	11.2 ± 10.1
800–820 °C (D)	−16.5 ± 1.2
820–840 °C	−42.5 ± 4.3
820–840 °C (D)	15.1 ± 1.2
820–840 °C (H14/D)	−34.8 ± 2.9

Data with “−” are index to compressive stress, Data without “−” are indicated to tensile stress

**Fig. 4:**
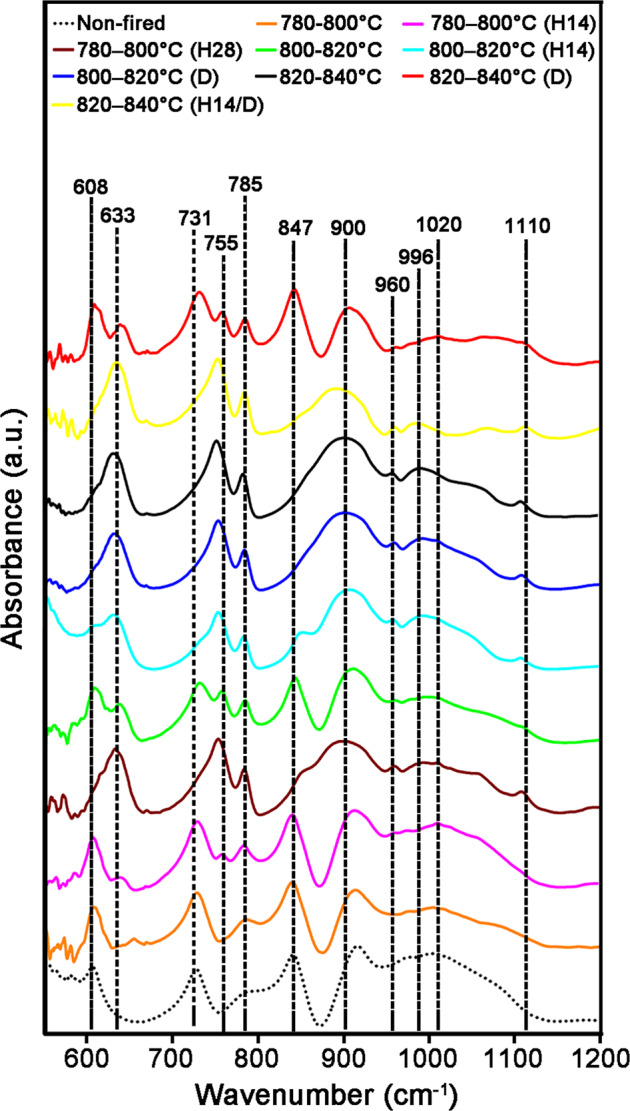
FTIR spectra of LDGC materials heated under various thermal conditions

As a supplement, the FTIR spectra of LDGC block exhibited various features which suggested that temperature, the holding time, and dual sintering could contribute to alterations in Si-related components (Fig. 4). Bands near to 731 and 847 cm^−1^ were attributed to LM [17, 18]. Peaks around 633, 755, 785, 1020 and 1110 cm^−1^ are intrinsic for LD [17–19]. The hump at 900 cm^−1^ was assigned to vibration of pyro-silicates (Si_2_O_7_)^6−^ as Q_1_ [20]. The kink at 996 cm^−1^ was caused by the vibration of SiO_2_ [21]. The fluctuation below 608 cm^−1^ was related to Li_3_PO_4_ [17]. Generally, the LM and Li_3_PO_4_ phases were detected in the non-fired, 780–800 °C, 780–800 °C (H14), 800–820 °C, 800–820 °C (H14), and 820–840 °C (D) groups. The characteristic peaks of LD were the primary components in the 780–800 °C (H28), 800–820 °C (D), 820–840 °C, and 820–840 °C (H14/D) groups. A prolonged holding time promoted the generation of the LD phase at 780–800 °C and showed stabilized effect on the LD phase at 820–840 °C during dual sintering. LD could be generated by dual sintering at a temperature below 820 °C but lose its phase stability when the LDGC was dual sintered above 840 °C, thus multiple crystal phases could be identified in 820–840 °C (D) group.

### Microstructural alteration

**Fig. 5:**
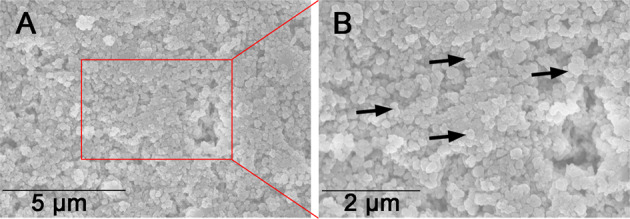
SEM images of representative morphologies of non-fired LDGC materials

**Fig. 6:**
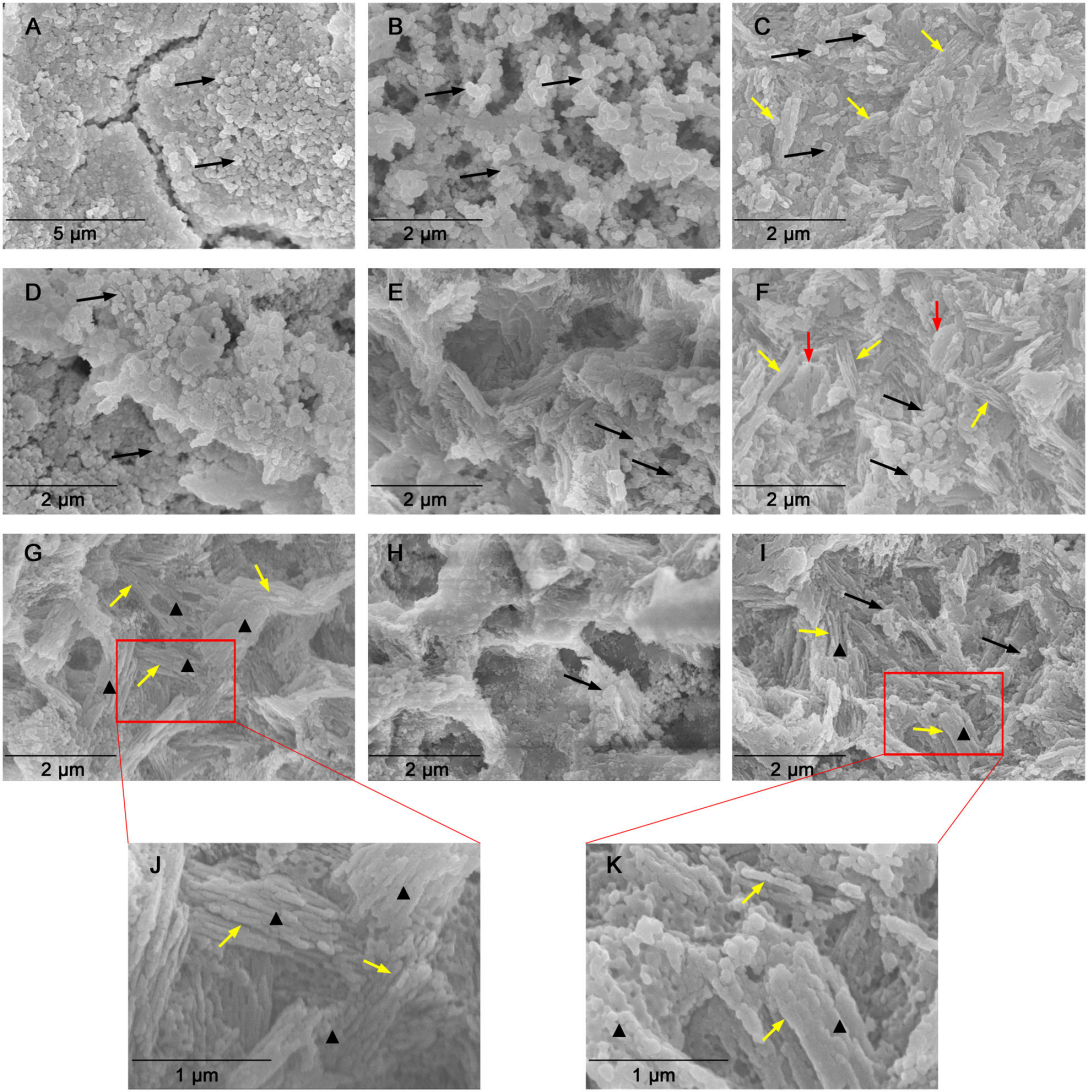
SEM images of representative morphologies of LDGC materials heated under various thermal conditions. **A** 780–800 °C; **B** 780–800 °C (H14); **C** 780–800 °C (H28); **D** 800–820 °C; **E** 800–820 °C (H14); **F** 800–820 °C (D); **G** 820–840 °C; **H** 820–840 °C (D); **I** 820–840 °C (H14/D); **J** Higher magnification of the rectangular region in **G**; **K** Higher magnification of the rectangular region in **I**. Black arrows denote crystal grains of small sizes. Yellow arrows denote fine crystal rods with clear grain boundaries. Red arrows denote melted crystal rods with large sizes. Black triangle denote crystal bundles

Before firing, the fine crystal grains were compressed to form a dense structure (Fig. 5A), “platelet”-like crystal grains of uniform diameter 0.2–0.3 μm were dispersed homogeneously in the LDGC block (Fig. 5B). Then, the crystal grains aggregated locally after firing at 780–800 °C, which led to cracks separating the stacked grains (Fig. 6A). When the holding time was prolonged to 14 min at 780–800 °C, the fine grains gathered as small clusters and a porous mesh structure was formed as well (Fig. 6B). In the 780–800 °C (H28) group, interlocking rods of length 0.5–0.7 μm became the predominant morphology, and scattered fine crystal grains (~0.3 µm) were identified (Fig. 6C). Apparently, prolonged holding time facilitated the generation of crystal rod at low sintering temperature. In the 800–820 °C group, the platelets appeared to be confluent to form large clusters (Fig. 6D). With the extension of holding time to 14 min, nascent crystal rods (~0.7 µm) with several voids and an embryonic form of an interlocked microstructure could be identified (Fig. 6E). Enlarged crystal rods (0.7–0.9 µm) with a clear profile could be recognized in the 800–820 °C (D) group, whereas a few fine grains (~0.3 µm) assembled locally and large crystal plates were identified (Fig. 6F). Therefore, at 800–820 °C, prolonged holding time could gently promote the generation of crystal rods, however, the reaction caused by dual sintering seemed to be rapid but beyond control. Uniform crystal rods of length 0.7–0.9 µm and a fully interlocked microstructure were the defining characteristics of LDGC material treated by a standard firing protocol (Fig. 6G). At 820–840 °C, dual sintering caused a melted appearance of crystal rods and increasing voids in the LDGC material (Fig. 6H), but a few fine crystal grains and fewer voids could be observed in the 820–840 °C (H14/D) group (Fig. 6I). Compared 820–840 °C group and 820–840 °C (H14/D) group at higher magnification (Fig. 6J, K), it was worth to note that the grain boundary of crystal rod could be identified from the interlocked crystal bundle.

Consistent with the SEM results, XPS, XRD and FTIR also highlighted that the LD phase was the predominant component in LDGC when the holding time was prolonged enough below 800 °C, showing corresponding characteristic spectra (Figs. 2, 3D, 4 and Table 3). Dual sintering was more conductive to accelerate the formation of LD phase than the prolonged holding time below 820 °C (Figs. 3F, G, 4 and Table 3). Above 840 °C, despite the XRD spectra were similar among these groups (Fig. 3H, F, J), significant phase transition and microstructural alteration occurred in dual sintered samples (Figs. 4, 6I and Table 3) whereas standard and prolonged holding time groups shared similar crystal phase and microstructure (Figs. 4, 6J, K and Table 3). LDGC groups, which contained more than 60% LD crystal phase (Table 3), obtained residual compressive stress to enhance their mechanical properties (Tables 2 and 4).

### Biocompatibility

**Fig. 7:**
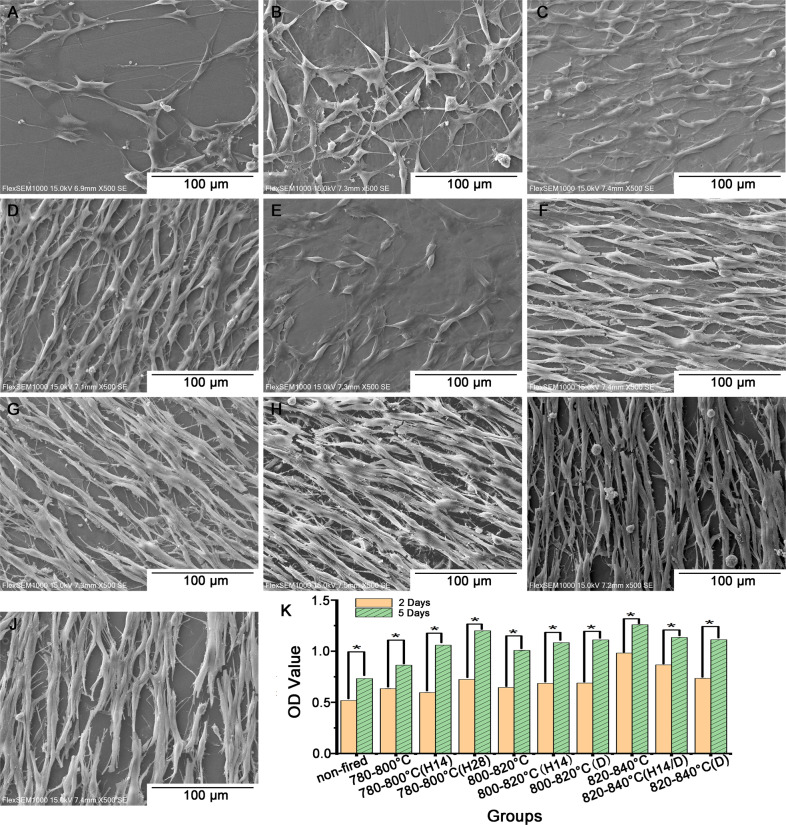
Representative SEM micrographs of MC3T3-E1 cells after 5 days culture on LDGC discs (**A**) non-fired; (**B**) 780–800 °C; (**C**) 780–800 °C (H14); (**D**) 780–800 °C (H28); (**E**) 800–820 °C; (**F**) 800–820 °C (H14); (**G**) 800–820 °C (D); (**H**) 820–840 °C; (**I**) 820–840 °C (D); (**J**) 820–840 °C (H14/D). SEM imaging parameters: EHT voltage level = 15.00 kV; magnification = ×500. **K** Proliferation activities of MC3T3-E1 cells on LDGC groups with various treatment as analyzed by CCK-8. Asterisks (∗) denoted statistically significant differences between 2 days and 5 days of culture (*P* < 0.05)

As shown in Fig. 7, MC3T3-E1 cells grown on LDGC discs for 5 days displayed an elongated spindle-shaped morphology in 780–800 °C (H28), 800–820 °C (H14), 800–820 °C (D), 820–840 °C, 820–840 °C (H14/D) and 820–840 °C (D) groups. By contrast, cells in 780–800 °C, 780–800 °C (H14), 780–800 °C (H28) and 800–820 °C groups appeared to be considerably elongated and flattened. The CCK‑8 assay results (Fig. 7K) revealed that the proliferation of MC3T3-E1 cells were significantly increased in all groups after 5 days of culture, although the OD values of 780–800 °C, 780–800 °C (H14) and 800–820 °C groups were slightly lower than that of the other groups. These results confirmed that the LDGC material offered outstanding biocompatibility during various treatments.

## Discussion

We demonstrated that dual sintering and holding time had a significant impact on regulating the mechanical properties and microstructure of LDGC material, and the alterations could be regulated by the temperature as well. Little negative impacts on biocompatible stability of LDGC material emanated from the fluctuation of these heating parameters. The corresponding alterations in mechanical properties were highly relevant to microstructure evolution, including surficial chemical alteration, crystal phases, residual stress and morphology.

The way that LD crystals strengthen the glass-ceramic is thought to aid the formation of an interlocking structure with rational crystal size and shape of LD [7]. Well-sintered LDGC could also present residual compressive stress to strengthen the toughness of the material [22]. Simba et al. [23] observed a glass-ceramic material comprising multiple phases with inferior mechanical properties formed at 820 °C. In contrast, a good-performance glass-ceramic material containing a primary LD crystal phase was generated at 840 °C. Consequently, a high proportion of LD phase coupled with homogeneous crystal grains and residual compressive stress could endow LDGC material with excellent mechanical properties, which our control group supported, named the 820–840 °C group.

Studies have shown that increasing the number of firing cycles adversely affects the mechanical properties of LDGC materials [11, 24]. Those results are in accordance with our data, which showed that the hardness and Young’s modulus deteriorated significantly after dual sintering at 840 °C (Fig. 1 and Table 2). Under this thermal condition, the microstructure (Fig. 6H) was similar to the disappearance of mesh structure and formation of a porous surface reported by Ozdogan et al., who demonstrated that multiple firing cycles negatively affected the mechanical properties of LDGC materials [24]. Melted appearance of crystal rods, which was showed in 820–840 °C (D) group (Fig. 6H), indicated the formation of large crystal-size LD grains which were less able to dissipate external stress, thus counteract the interlocking effect to cause a degradation of the strength [25, 26]. Furthermore, a decrease in the XRD peaks of the LD phase was observed when the number of firing cycles was increased [11], and inappropriate thermal treatment could result in reversal of transformation from the LM phase to LD phase [27]. Therefore, reduction of the LD phase and the reappearance of a glassy-phase signal in 820–840 °C (D) group could be explained (Table 3 and Figs. 2–4). Such a transformation of crystal phase generated the residual tensile stress effect, which facilitated the crack propagation in the glass matrix [22, 25], resulting in a dramatic decrement in the mechanical properties of LDGC. It should be noted that crystal grains with large sizes could be identified in dual sintering groups (Fig. 6F, H), and the heterogeneous crystal phase was characterized in the 820–840 °C (D) group (Fig. 4). Both of them could contribute to a mismatch of the crystal size and thermal expansion coefficient (TEC). The residual tensile stress effect was generated as a drawback for the mechanical properties of the LDGC material [22].

The threshold temperature for crystallization of the LD phase is ~820 °C in short-term heat treatment, which indicates that treatment under such conditions is insufficient for phase transformation [28]. The extension of the holding time does not promote the transformation of LM crystals to LD crystals below 820 °C [7, 23]. However, small size LD crystals were detected in 780–800 °C groups when the holding time was prolonged enough, together with the strengthening of the material’ mechanical properties (Table 2). Our results suggested that the prolonged holding time contributed less to increasing the crystal grain size but gently promoted the phase transformation reaction below 820 °C. Although sintering at a low temperature could limit the growth of LD crystals, β-cristobalite consumption continued during the formation of LD crystals [7, 29]. Quenching of the 731 and 847 cm^−1^ peaks upon extension of the holding time, as shown in 780–800 °C (H28) group (Fig. 4), indicated a decline in the content of the glassy matrix in the LDGC material, which, in turn, promoted residual compressive stress to enhance the mechanical properties of the LDGC block. Prolonged holding time not only promoted the growth of LD crystal at low temperature but also stabilized the crystal phase (Table 3) and grain boundary of LD (Fig. 6K) at high temperature. Interestingly, unlike dual sintering alone, dual sintering with a prolonged holding time at 820 °C–840 °C caused a slight decrease in the mechanical properties of the LDGC material. It has been reported that a prolonged holding time at 840 °C can significantly improve Young’s modulus and hardness of a glass ceramic material [6, 30], which can be attributed to the further crystallization of LD [27]. We note that a longer holding time facilitated a further phase transformation without increasing the crystal size significantly at 820 °C–840 °C (Fig. 6K), after which the external stress could be cushioned. Moreover, high crystallized volume fraction as LD phase (Table 3) could be another reason for inducing residual compressive stress (Table 4), by which crack propagation could be resisted [22]. Prolongation of the holding time could increase the percentage of the LD phase, whereas crystals of small size were identified (Fig. 6I). The sporadic small particles may trigger local stress concentration to weaken the LDGC that is treated with 820 °C–840 °C (H14/D).

For our case, the mechanical properties of LDGC were reduced when treated with the heating schedules of 780–800 °C (H28) and 800–820 °C (D), even though the crystal phase and microstructure in both groups were close to 820–840 °C group. These results appeared to be in good agreement with previous studies. Heat treatment at a low temperature resulted in multi-phase crystallization for Li_3_PO_4_, β-cristobalite, and LD, and the LM phase to be retained [31]. The heterogeneity of the TEC of crystal phases can reduce the stored elastic energy available for the creation or propagation of cracks [22, 32]. Crystallization of LD continued during the second firing cycle thanks to consumption of the amorphous phase and LM crystals (Table 3) below 820 °C, but the neterogeny of crystal grain size (Fig. 6F) in 800–820 °C (D) group could alter the residual stress effect, which could counteract the interlocking effect to cause a degradation of the strength [22, 25, 26].

**Fig. 8:**
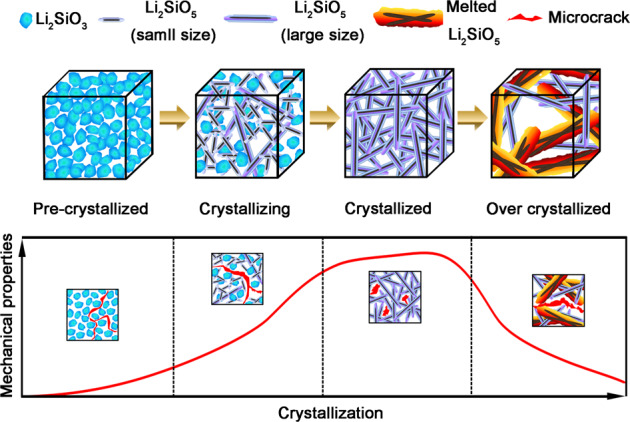
Schematic of microstructural and mechanical evolution of LDGC materials during thermal processing. The gap between LM grains, small size LD grains and melted LD grains facilitates the propagation of microcrack, thus the mechanical properties of LDGC were decreased. However, dense interlocking structure confines the areas of cracks

In summary Fig. 8 illustrates the possible evolution of phase transformation and microstructure, as well as the possible mechanism of microcrack propagation in each crystallization stage of LDGC. Crystal phase transformation and crystal size are highly relevant to the final microstructure of LDGC, which in turn regulate the mechanical properties of LDGC.

## Conclusions

An interlocking structure with homogeneity of crystal phase and size facilitated the strengthening of mechanical properties of LDGC material. Dual sintering alone at 840 °C caused adverse alterations in the microstructure and crystal phase of LDGC, as well as transferred residual stress from compression to tension, resulting in inferior mechanical properties of materials. A prolonged holding time promoted the crystallization and stability of LD crystal grains without increasing the grain size significantly, and showed an antagonistic effect to the mechanical-property weakening of the LDGC material caused by dual sintering alone at 840 °C. For extending the restoration longevity of LDGC, prolongation of the holding time at the second stage of a standard firing cycle could be considered when dual sintering is inevitable or predictable.
